# Association between sleep duration and CVD mortality: a prospective cohort study based on middle-aged and elderly chest pain patients

**DOI:** 10.1186/s12872-025-05252-z

**Published:** 2025-11-26

**Authors:** Congcong Lv, Yingying Li, Xue Zhang, Xiaolan Zhang, Jianmin Qu, Xiaoxu Ren, Yong Liu

**Affiliations:** 1 Hematopoietic Stem Cell Transplantation Center, State Key Laboratory of Experimental Hematology, National Clinical Research Center for Blood Diseases, Hai he Laboratory of Cell Ecosystem, Institute of Hematology and Blood Diseases Hospital, Chinese Academy of Medical Sciences and Peking Union Medical College, 300020 Tianjin, China; 2 Tianjin Institutes of Health Science, Tianjin, 301600 China; 3 Department of Neurology, Third People’s Hospital of Liaocheng, Liaocheng, Shandong Province, lii605@163.com, , 252000, shandong, , China; 4 Department of Otolaryngology-Head and Neck Surgery, Affiliated Hospital of Jining Medical University, Shandong, 272002 China; 5 Department of Intensive Care Unit, Tongxiang First People’s Hospital, Zhejiang Province, 314500 Tongxiang, China; 6 Department of Anesthesiology, The Affiliated Hospital of Qingdao University, Shinan District, mzysly@126.com, No.16 of Jiangsu Road, 266003 Qingdao, China

**Keywords:** Sleep durations, CVD mortality, Chest pain patients, NHANES

## Abstract

**Background:**

Previous studies have yielded varying conclusions about the relationship between sleep duration and cardiovascular disease (CVD) mortality across different populations. Therefore, it is crucial to examine this relationship specifically within the U.S. chest pain population.

**Objective:**

This study aims to evaluate the association between sleep duration and cardiovascular disease (CVD) mortality among a U.S. population presenting with chest pain.

**Methods:**

This prospective cohort study included 70,190 participants from the 2005–2018 National Health and Nutrition Examination Survey (NHANES). Participants who reported ever experiencing chest pain or discomfort, with severe chest pain lasting more than half an hour, were categorized as having chest pain. Mortality data were obtained by linking the cohort database with the National Death Index as of December 31, 2018. Cardiovascular disease (CVD) mortality was classified according to the 10th revision of the International Classification of Diseases (ICD-10) and included the following codes: I00-I09 (acute rheumatic fever, chronic rheumatic heart disease), I11 (hypertensive heart disease), I13 (hypertensive heart and kidney disease), I20-I25 (ischemic heart disease), I26-I28 (pulmonary embolism and other acute pulmonary heart disease), I29, I30-I51 (other forms of heart disease), and I60-I69 (cerebrovascular disease). Data were analyzed between June and July 2024.

**Results:**

Among 2,952 US patients with chest pain, the mean age was 57.92 ± 11.63 years, with 1,424 males (49.01%). A total of 1,439 participants (48.74%) reported sleeping ≤ 6 h, while 376 participants (12.73%) reported sleeping > 9 h. After a median follow-up of 85.92 months, there were 164 CVD-related deaths. Compared with patients who had normal sleep duration, those with insufficient sleep had an adjusted hazard ratio (HR) of 1.99 (95% confidence interval [CI], 1.36–2.89; *P* < 0.001) for CVD mortality, while those with excessive sleep had an adjusted HR of 2.39 (95% CI, 1.37–4.16; *P* = 0.002). Sensitivity analyses, which excluded patients who died within 2 years of follow-up (sleep duration ≤ 6 h: HR, 1.94; 95% CI, 1.31–2.87; sleep duration > 9 h: HR, 2.40; 95% CI, 1.29–4.44) or those with a baseline history of cancer (sleep duration ≤ 6 h: HR, 1.98; 95% CI, 1.27–3.07; sleep duration > 9 h: HR, 2.86; 95% CI, 1.51–5.41), demonstrated that the association between sleep duration and CVD mortality remained robust. Further exclusion of patients with incomplete data (sleep duration ≤ 6 h: HR, 2.23; 95% CI, 1.51–3.30; sleep duration > 9 h: HR, 2.47; 95% CI, 1.35–4.53) also supported these findings, indicating a consistent relationship between sleep duration and CVD mortality among US chest pain patients.

**Conclusions:**

Both longer and shorter sleep durations are associated with increased cardiovascular disease mortality in the U.S. chest pain population.

**Supplementary Information:**

The online version contains supplementary material available at 10.1186/s12872-025-05252-z.

## Introduction

Chest pain is one of the most common major complaints in the emergency department (ED), with a prevalence ranging from 5 to 12% [1]. The American Heart Association predicts that clinical cardiovascular disease (CVD) will affect over 45 million adults by 2050. Additionally, CVD, including hypertension, is expected to impact more than 184 million adults by that time, representing over 61% of the adult population [2]. This anticipated increase in the prevalence of cardiovascular risk factors and diseases over the next 30 years underscores the need for effective clinical and public health interventions [3].

Sleep is a significant health risk factor [4]. Previous studies have highlighted the potential benefits of optimizing sleep duration for the primary prevention of cardiovascular disease in the U.S. adult population. While there is limited evidence on the association between sleep disorders and CVD mortality [5], other research has confirmed a correlation between sleep disorders and chest pain [6]. Both excessive sleep and insufficient sleep have been linked to an increased risk of chest pain. Sleep disorders are recognized as important social determinants of health in the chest pain population and are associated with cardiovascular risk factors, as well as CVD events and mortality [3, 7]. Despite this, few studies have specifically examined the relationship between sleep duration and CVD mortality in patients with chest pain. This study is significant in its potential to address and potentially reverse CVD mortality associated with sleep duration.

Therefore, we utilized a large follow-up cohort from the National Health and Nutrition Examination Survey (NHANES) to assess the association between sleep duration and cardiovascular disease (CVD) mortality in individuals with chest pain.

## Methods

### Study population

NHANES, conducted by the National Center for Health Statistics (NCHS) (www.cdc.gov/nchs/nhanes/about_nhanes.htm), assesses the health and nutritional status of the U.S. population. It employs a complex, multistage, multilevel probability sampling methodology to select nationally representative samples [3]. The survey comprises two parts: a home interview and a physical examination conducted at a mobile examination center (MEC).

**Fig. 1:**
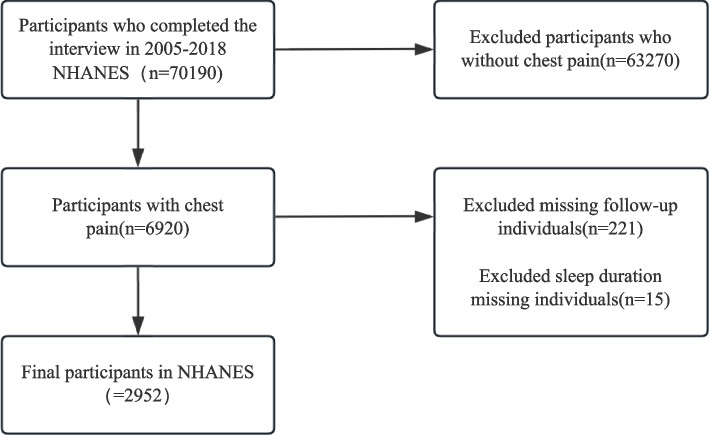
Flow chart of patient disposition

For this cohort study, data from adult subjects (≥ 20 years of age) across the 2005–2018 NHANES cycles were utilized. The cardiovascular health questionnaires from these cycles were specifically used to identify participants who reported having ever experienced chest pain or discomfort, or severe chest pain lasting more than half an hour. Exclusion criteria included missing follow-up data, incomplete fasting weights, absent sleep duration records, and missing covariate information. A flowchart of participant enrollment is shown in Fig. 1.

### Ethics approval

The research protocol was approved by the NCHS Ethics Review Committee (https://www.cdc.gov/nchs/nhanes/about/erb.html?CDC_AAref_Val=https://www.cdc.gov/nchs/nhanes/irba98.htm), and all participants provided informed consent prior to their involvement in the study. Secondary analyses did not require additional institutional review board approval. Clinical trial number: not applicable.

### Assessment of sleep duration

Sleep duration data were collected through health interviews using a self-report questionnaire (SLD010H). Participants answered the computer-assisted personal interview question, "How much sleep do you usually get on weekdays or weeknights?" Based on previous research, this study categorized sleep duration into three groups: short (6 h or less per night), normal (7 to 9 h per night), and long (more than 9 h per night) [6, 8].

### Assessment of chest pain

During the health interview, subjects were asked, "Do you feel any pain or discomfort in your chest?" Based on previous studies [6], this study categorized chest pain into painful and painless. The population included in this study consisted of patients with chest pain. According to the 2021 American College of Cardiology Guidelines [9], chest pain encompasses more than just pain; it also includes pressure, tightness, or discomfort in the chest, shoulders, arms, neck, back, upper abdomen, or jaw, as well as symptoms such as shortness of breath and fatigue that could indicate angina.

### Outcome definitions (CVD Mortality)

Mortality data were obtained by linking the cohort database to the Centers for Disease Control's National Death Index as of December 31, 2018. According to the 10th revision of the International Classification of Diseases (ICD-10), CVD mortality includes the following disease codes: 100–109 (acute rheumatic fever, chronic rheumatic heart disease), 113 (hypertensive heart and kidney disease), 120–125 (ischemic heart disease), 126–128 (pulmonary embolism and other acute pulmonary heart diseases), 129 (cardiovascular disease of various causes), 130–151 (other forms of heart disease), and 160–169 (cerebrovascular disease).

### Assessment of covariates

Assessment of covariates was based on previous research [10]. Potential covariates included age, gender [11], marital status, race/ethnicity, education level, household income, body mass index (BMI), tobacco use, alcohol use, chronic disease status (including dyslipidemia, hypertension, diabetes, neoplasia, and CVD) [12], general health, and exercise [13, 14]. Participants were categorized into two groups based on marital status: living alone (unmarried, separated, divorced, or widowed) and living with a partner (married) [6]. Education levels were divided into two categories: ≤ 12 years and > 12 years [15]. Smoking status was categorized as never smoked, current smoker, and former smoker [16]. Drinking status was classified as never drank (fewer than 12 drinks in a lifetime) and drank (12 or more drinks in any given year). Vigorous physical activity was categorized as yes or no, based on whether the participant engaged in strenuous activity, fitness, or recreational activities that significantly increased respiration or heart rate [17, 18]. General health was categorized into three levels: very good, good, and fair or poor [15]. Chronic diseases were classified as either absent or present, with presence defined as having one or more of dyslipidemia, hypertension, diabetes mellitus, neoplasia, or CVD [16, 19].

### Statistical analysis

This study represents a secondary analysis of publicly available data. For the NHANES dataset, it is crucial to use sampling weights and sampling design variables to avoid biased estimates and inflated significance levels. According to NHANES analytic guidelines, fasting weights should be used for analyses. The National Death Index, updated every four years, provided the most recent follow-up data as of December 31, 2018. Consequently, the follow-up period for each participant was calculated from the date of the MEC test until the date of death or the end of follow-up (December 31, 2018).

Categorical and continuous variables are reported as unweighted values (weighted percentages) and means (standard deviations), respectively. Linear regression analysis and chi-square tests were employed to compare continuous and categorical variables. Multifactorial Cox proportional hazards regression models were used to determine the hazard ratio (HR) and 95% confidence interval (95% CI) for the relationship between sleep duration and CVD mortality among patients with chest pain. Model Ⅰ was unadjusted, Model Ⅱ was adjusted for age, sex, and NHANES cycle, and Model Ⅲ was adjusted for age, gender, BMI, race, education, marital status, smoking, alcohol use, exercise, chronic disease, and general health status. Additionally, we conducted several sensitivity analyses to assess the robustness of our results. First, to address potential reverse causality, we excluded patients with less than 2 years of follow-up. Second, we removed participants with a history of cancer, as cancer could influence mortality rates. Third, we analyzed the impact of excluding patients with missing data. All adjustment models were refined using the full set of covariates.

Analysis was conducted using R version 4.2.2 (http://www.R project.org; The R Foundation, Vienna, Austria) and Free Statistics software version 1.9.2 (Beijing Free Clinical Medical Technology Co., Ltd, Beijing, China) [20]. Statistical significance was defined as a two-sided p-value less than 0.05. Data analysis was performed between March and May 2024.

## Results

### Baseline characteristics

Data from seven NHANES cycles (2005–2006, 2007–2008, 2009–2010, 2011–2012, 2013–2014, 2015–2016, and 2017–2018) were utilized in this study. Among the 70,190 participants, 6,920 had reported chest pain. After excluding individuals with missing follow-up data (*n* = 221), missing information on sleep duration (*n* = 15), and missing fasting weights (*n* = 3,741), a total of 2,952 individuals were included in the final analysis (see Fig. 1).

At baseline, a total of 1,439 participants (48.74%) reported sleeping 6 h or less per night, while 376 participants (12.73%) reported sleeping 9 h or more per night. The baseline characteristics of the 2,952 participants are detailed in Table 1. The mean age of participants was 57.92 (± 11.63) years, with 1,528 (50.99%) being male. Compared to participants with normal sleep duration (mean age 56.23 ± 11.13 years), those with short sleep (≤ 6 h) had a mean age of 58.03 (± 11.69) years, while those with long sleep (> 9 h) had a mean age of 62.52 (± 11.60) years.

**Table 1:** Characteristics of participants in the NHANES 2005–2018 cycles

Variable	All participants ^a^	Sleep Duration(hours)			
Questionnaire-Based Data	Total	≤ 6 h	7 h ~ 9 h	> 9 h	*P-Value*
No	2952	1439	1137	376	
Sex, n (%)					
Male	1424(49.01)	715 (50.88)	535 (47.64)	174 (44.79)	0.264
Female	1528 (50.99)	724 (49.12)	602 (52.36)	202 (55.21)	
Age(years)	57.92 (11.63)	58.03 (11.69)	56.21 (11.13)	62.52 (11.60)	< 0.001
BMI(kg/m^2^)	30.00 (7.02)	29.69 (6.72)	30.43 (7.36)	30.11 (7.22)	0.115
Race/ethnicity, n (%)					
Mexican American	1437 (72.74)	748(76.52)	490 (66.43)	199(74.68)	< 0.001
Hispanic	625(10.99)	255(8.32)	309 (15.79)	61(8.61)	
Non-Hispanic White	385 (5.65)	196 (5.98)	138 (5.13)	51(5.74)	
Non-Hispanic Black	281(4.40)	133 (4.00)	107(4.59)	41(5.58)	
Others	224 (6.21)	107 (5.17)	93(8.05)	24(5.38)	
Education level(years), n (%)					
≤ 12	1532(44.30)	699(39.58)	618(50.33)	215(47.40)	0.001
> 12	1420(55.70)	740(60.42)	519(49.67)	161(52.60)	
Marital status, n (%)					
Living alone	1723 (64.31)	900(69.39)	633(58.78)	190(58.14)	< 0.001
Married or living with a partner	1420 (35.69)	539(30.61)	504(41.22)	186(41.86)	
Drink status, n (%)					
No	1042(29.86)	480 (28.06)	408 (30.54)	154(35.79)	0.105
Yes	1910(70.14)	959(71.94)	729 (69.46)	222(64.21)	
Smoking status, n (%)					
Never	1329(43.25)	676(45.84)	488(39.65)	165(42.46)	0.003
Former	953(33.27)	477(34.33)	348(31.25)	128(34.52)	
Current	670(23.48)	286(19.83)	301(29.10)	83(23.01)	
Vigorous recreational activities, n (%)					
No	2578(84.06)	1212(79.85)	1013(87.62)	353(92.24)	< 0.001
Yes	374(15.94)	227(20.15)	124(12.38)	23(7.76)	
Current health status, n (%)					
Very good to excellent	614(26.32)	370(32.53)	173(17.97)	71(23.53)	< 0.001
Good	1147(42.09)	588(42.31)	436(42.47)	123(39.97)	
Poor to fair	1191(31.59)	481(25.16)	528(39.56)	182(36.50)	
Chronic diseases, n (%)					
No	519(21.41)	271(23.48)	185(18.43)	63(21.08)	0.127
Yes	2433(78.59)	1168(76.52)	952(81.57)	313(78.92)	
CVD, n (%)					
No	2790(95.81)	1382(97.18)	1060(94.42)	348(93.83)	0.001
Yes	162(4.19)	57(2.82)	77(5.58)	28(6.17)	
Follow-up time(months)	85.92 (50.18)	83.19 (49.38)	97.94 (49.05)	62.44 (46.94)	< 0.001

*Abbreviations*: *NHANES*, National Health and Nutrition Examination Survey, *BMI* Body mass index, *CVD* Cardiovascular disease

^a^Data are presented as unweighted number (weighted percentage) for categorical variables, and mean (median) for continuous variables

In terms of race/ethnicity, Mexican-Americans were the most represented group, comprising 1,437 participants (72.74%). Sleep disorders were more prevalent among those with higher levels of education, with 740 (60.42%) of those with short sleep and 161 (52.60%) of those with long sleep reporting sleep disturbances [21]. Notably, sleep disorders were also observed in individuals who lived alone, consumed alcohol, had never smoked, did not engage in vigorous physical activity, and those who reported good general health (Table 1).

We performed univariate logistic regression analysis (Supplementary Table 1) followed by multivariate logistic regression analysis to evaluate the relationship between covariates, Table 2 displays the association between different sleep durations and cardiovascular disease (CVD) mortality in patients with chest pain.

**Table 2:** Hazard ratios of CVD mortality by Sleep duration among adults in NHANES 2005–2018

Variable	Model Ⅰ (HR,95%CI)	*P*	Model Ⅱ (HR,95%CI)	*P*	Model Ⅲ (HR,95%CI)	*P*
Duration of sleep(h)						
7–9	Ref		Ref		Ref	
≤ 6	1.65(1.15, 2.36)	0.006	2.2(1.56,3.10)	< 0.001	1.99(1.36,2.89)	< 0.001
> 9	2.99(1.72, 5.20)	< 0.001	2.08(1.22,3.57)	0.008	2.39(1.37,4.16)	0.002

Model Ⅰ, not adjusted

Model Ⅱ, we adjusted age and sex

Model Ⅲ, was adjusted for Model Ⅱ plus BMI, Race/ethnicity, Education level, Marital status, Drink status, Smoking status, Vigorous recreational activities, Current health status, Chronic diseases

*Abbreviations*: *CI* Confidence interval, *HR* Hazard ratios, *CVD* Cardiovascular disease, *BMI* Body mass index

In Model I, the risk ratio for short sleep (≤ 6 h) compared to normal sleep (7–9 h) was 1.65 (95% CI, 1.15–2.36; *P* = 0.006), indicating that short sleep was associated with a significantly higher risk of CVD mortality. The risk ratio for long sleep (> 9 h) was 2.99 (95% CI, 1.72–5.20; *P* = 0.001), suggesting that longer sleep duration was also significantly associated with increased risk.

In Model II, which adjusted for age and sex, the risk ratio for short sleep increased to 2.20 (95% CI, 1.56–3.10; *P* < 0.001), further highlighting the increased risk associated with shorter sleep duration after accounting for these variables. The risk ratio for long sleep decreased to 2.08 (95% CI, 1.22–3.57; *P* = 0.008).

In Model III, which adjusted for a full range of variables including age, sex, BMI, race, education, marital status, smoking, alcohol use, exercise, chronic disease, and general health status, the risk ratio for short sleep slightly decreased to 1.99 (95% CI, 1.36–2.89; *P* < 0.001). This indicates that shorter sleep duration remained significantly associated with higher health risks even after comprehensive adjustment. The risk ratio for long sleep was further adjusted to 2.39 (95% CI, 1.37–4.16; *P* = 0.002), demonstrating that the association between longer sleep duration and increased risk of CVD mortality remained significant even after controlling for additional variables.

### Curve fitting and inflection point analysis

We used restricted cubic spline (RCS) to construct smooth curves and explore the relationship between different sleep durations and cardiovascular disease (CVD) mortality in patients with chest pain (Supplementary Fig. 1), which revealed a nonlinear association between these variables. The threshold effect was assessed using segmented linear regression (Table 3).

**Table 3:** The result of two-piecewise linear regression mode

Threshold of sleep duration	HR, 95%CI	*P*
Sleep duration < 7 h	0.75(0.61, 0.91)	0.005
Sleep duration ≥ 7 h	1.38(1.12, 1.70)	0.003
P for log likelihood ratio test		< 0.001

Fully adjusted models account for age, sex, BMI, race/ethnicity, education level, marital status, drinking status, smoking status, vigorous recreational activities, current health status, and chronic diseases

*Abbreviations*: *CI* Confidence interval, *HR* Hazard ratios, *CVD* Cardiovascular disease, *BMI* Body mass index

The results indicated that CVD mortality was inversely associated with sleep duration up to 7 h, with a hazard ratio (HR) of 0.75 (95% confidence interval [CI], 0.61–0.91; *P* = 0.005). However, when sleep duration was 7 h or more, the incidence of CVD mortality increased with longer sleep duration, showing an HR of 1.38 (95% CI, 1.12–1.70; *P* = 0.003). Therefore, around 7 h of sleep was identified as the duration associated with the lowest incidence of CVD deaths.

### Survival analysis

**Fig. 2:**
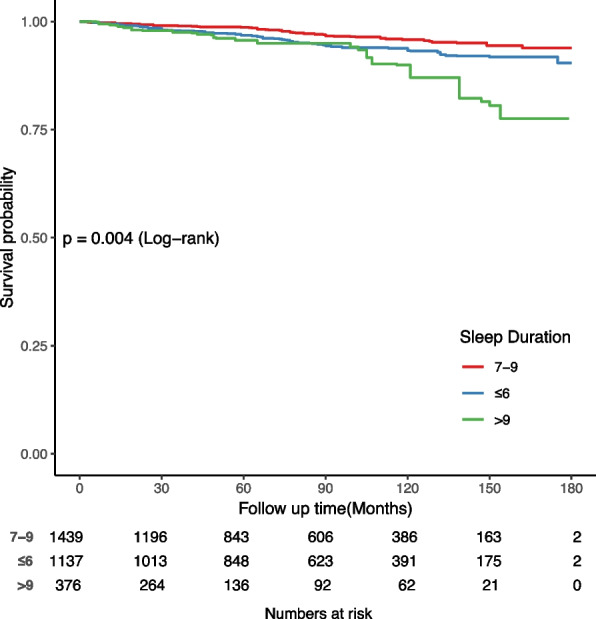
Kaplan–Meier survival curves describing the relationship between sleep duration and CVD mortality in chest pain patients

Among the 2,952 participants, with a mean follow-up duration of 85.92 months (median follow-up of 50.18 months, ranging from 1 to 180 months), a total of 162 cardiovascular disease (CVD) deaths were observed. Kaplan–Meier curves demonstrated a significantly higher CVD mortality rate among individuals with either insufficient or excessive sleep compared to those with normal sleep duration (log-rank test, *P* < 0.001) (Fig. 2).

### Subgroups and sensitivity analysis

In stratified analyses, the association between sleep duration and CVD mortality remained consistent across different age groups, marital statuses, sexes, education levels and CVD (past medical history) (Supplementary Fig. 2). Sensitivity analyses confirmed the robustness of this association, showing that the significant correlation between sleep duration and CVD mortality persisted even after adjusting for baseline characteristics and comorbidities. This correlation remained stable when excluding participants with less than 2 years of follow-up, those with a history of cancer at baseline, and patients with missing covariates. These findings are detailed in Table 4. Additionally, we conducted a subgroup analysis after excluding participants with a history of CVD at baseline to assess the relationship between sleep duration and cardiovascular mortality. We were pleased to find that the results remained consistent and robust even after excluding these participants, as shown in Supplementary Fig. 3.

**Table 4:** Sensitivity analyses

Variable	Duration of sleep (h)		
	≤ 6	7 ~ 9	> 9
Patients with a follow-up time of less than 2 years			
Crude model	1.60 (1.07, 2.40)	Ref	2.99(1.64, 5.44)
Adjusted model	1.94 (1.31, 2.87)	Ref	2.40(1.29, 4.44)
Exclude patients with tumors			
Crude model	1.81(1.18, 2.78)	Ref	3.89(2.08, 7.27)
Adjusted model	1.98(1.27, 3.07)	Ref	2.86(1.51, 5.41)
Delete patients with missing data			
Crude model	1.80(1.21, 2.68)	Ref	2.23(1.51, 3.30)
Adjusted model	3.21(1.74, 5.92)	Ref	2.47(1.35, 4.53)

Fully adjusted models account for age, sex, BMI, race/ethnicity, education level, marital status, drinking status, smoking status, vigorous recreational activities, current health status, and chronic diseases

*Abbreviations: CI* Confidence interval, *HR* Hazard ratios, *CVD* Cardiovascular disease, *BMI* Body mass index

## Discussion

To the best of our knowledge, this large NHANES prospective cohort study clearly demonstrates that both insufficient and excessive sleep are independently associated with an increased risk of cardiovascular disease (CVD) mortality among patients with chest pain. Our subgroup analyses did not reveal any significant interactions between these associations and demographic or clinical variables. These findings underscore the critical clinical implications of sleep duration in the management of patients with chest pain.

Previous research has established a link between self-reported sleep duration and CVD mortality. Zhao et al. reported that the highest association with all-cause and CVD mortality was observed with an objective sleep duration of 5 h or less. They found that both short (≤ 4 h) and long (> 8 h) sleep durations were associated with an increased risk of all-cause and CVD mortality [20]. Similarly, among patients with type 2 diabetes mellitus, both short and long sleep durations (≤ 5 h and ≥ 10 h) were strongly associated with an elevated risk of cardiovascular disease morbidity and mortality compared to the reference of 7 h of sleep [22].

Appropriate physical activity may mitigate the adverse effects of both short and long sleep durations on mortality risk [13]. Analysis of five cycles of NHANES data from 2005 to 2014 highlighted a strong association between sleep problems and increased mortality risk, suggesting that individuals with a history of cardiovascular disease or cancer may be at higher risk and warrant more intensive interventions [3]. Our findings align with this, as the lowest risk of all-cause and CVD mortality was observed with approximately 7 h of sleep [3].

Furthermore, our study supports the conclusion that sleep duration is an independent risk factor for cardiovascular disease mortality and morbidity, consistent with findings in women from previous research. Additionally, our results did not show significant differences between male and female participants. Studies have also indicated that married individuals or those living with a partner report fewer symptoms of insomnia compared to single or solitary individuals, a finding consistent with our results [23].

Sleep duration and insomnia are known to influence the pathophysiology of cardiovascular disease through inflammatory [24], autonomic, and metabolic pathways. These physiological mechanisms help explain the observed relationship between sleep and cardiovascular disease. For example, short sleep duration is significantly associated with elevated levels of circulating interleukin 6 (IL-6), while long sleep duration is linked with increased IL-6 and C-reactive protein levels [25].

This study provides clear insights into the relationship between sleep duration and cardiovascular mortality risk among patients with chest pain, using data from the NHANES database. Our large, prospective, population-based cohort study, which includes a substantial number of outcome cases and a lengthy follow-up period, offers robust statistical power for subgroup analyses. By excluding participants with less than 2 years of follow-up, those with a history of cancer at baseline, and patients with missing covariate data, we aimed to reduce potential biases and confounding factors affecting our results. The comprehensive approach of our study enhances its relevance and provides strong evidence for the impact of sleep duration on cardiovascular death risk among chest pain patients.

However, this study also has limitations. As an observational study, it cannot establish a causal relationship between sleep duration and cardiovascular mortality risk. Additionally, the NHANES questionnaires were based on a single assessment, which may introduce recall bias and potentially underestimate the strength of the observed associations. We focused primarily on patients who reported symptoms of chest pain, without categorizing the specific types of chest pain. Moreover, our study’s findings are limited to participants from the United States, which may affect the generalizability of the results to other populations. To address these limitations, future multicenter controlled trials are needed to validate and extend our findings.

## Conclusions

Among U.S. patients with chest pain, both insufficient and excessive sleep were significantly associated with an increased risk of cardiovascular disease mortality, with an optimal sleep duration identified as 7 h. These findings are significant and underscore the need for further validation and confirmation to solidify the relationship between sleep duration and cardiovascular outcomes.

## Supplementary Information

Supplementary Material 1: Supplementary Figure 1: Sleep Duration Inflection Point Diagram. *Abbreviations*: CI, confidence interval; CVD, cardiovascular disease; HR, hazard ratio.

Supplementary Material 2: Supplementary Figure 2：Forest plot of multivariable logistics analysis between sleep duration and CVD mortality. Note: The stratifications were adjusted for all variables（age, sex, BMI, Race/ethnicity, Education level, Marital status, Drink status, Smoking status, Vigorous recreational activities, Current health status, Chronic diseases）except for the stratification factor itself. Squares represent the HRs and horizontal lines represent 95% CIs. Diamonds represent the overall HR, and the outer points of the diamonds represent the 95% CI. *Abbreviations*: BMI, body mass index; CI, confidence interval; CVD, cardiovascular disease; HR, hazard ratio; NHANES, National Health and Nutrition Examination Survey.

Supplementary Material 3: Supplementary Figure 3:Forest plot of multivariable logistics analysis between Hazard ratios of CVD mortality by Sleep duration among. *Abbreviations*: HR, hazard ratio; CI, confidence interval; CVD, cardiovascular disease.

Supplementary Material 4.Supplementary Table 1. Association of covariates and CVD mortality. Abbreviations: CI, confidence interval; OR, odds ratio.

## Data Availability

The data that support the findings of this study are available from the corresponding author Yong Liu upon reasonable request. Public datasets used in this study are available online. The repository names and accession numbers are available at NHANES—National Health and Nutrition Examination Survey Homepage (cdc.gov) (accessed: March 28, 2024).
