# Supplementary figures and images for: Association between sleep duration and CVD mortality: a prospective cohort study based on middle-aged and elderly chest pain patients
Source: BMC Cardiovasc Disord. 2025 Nov 26;25:846. doi: 10.1186/s12872-025-05252-z (PMC12659272; doi:10.1186/s12872-025-05252-z)

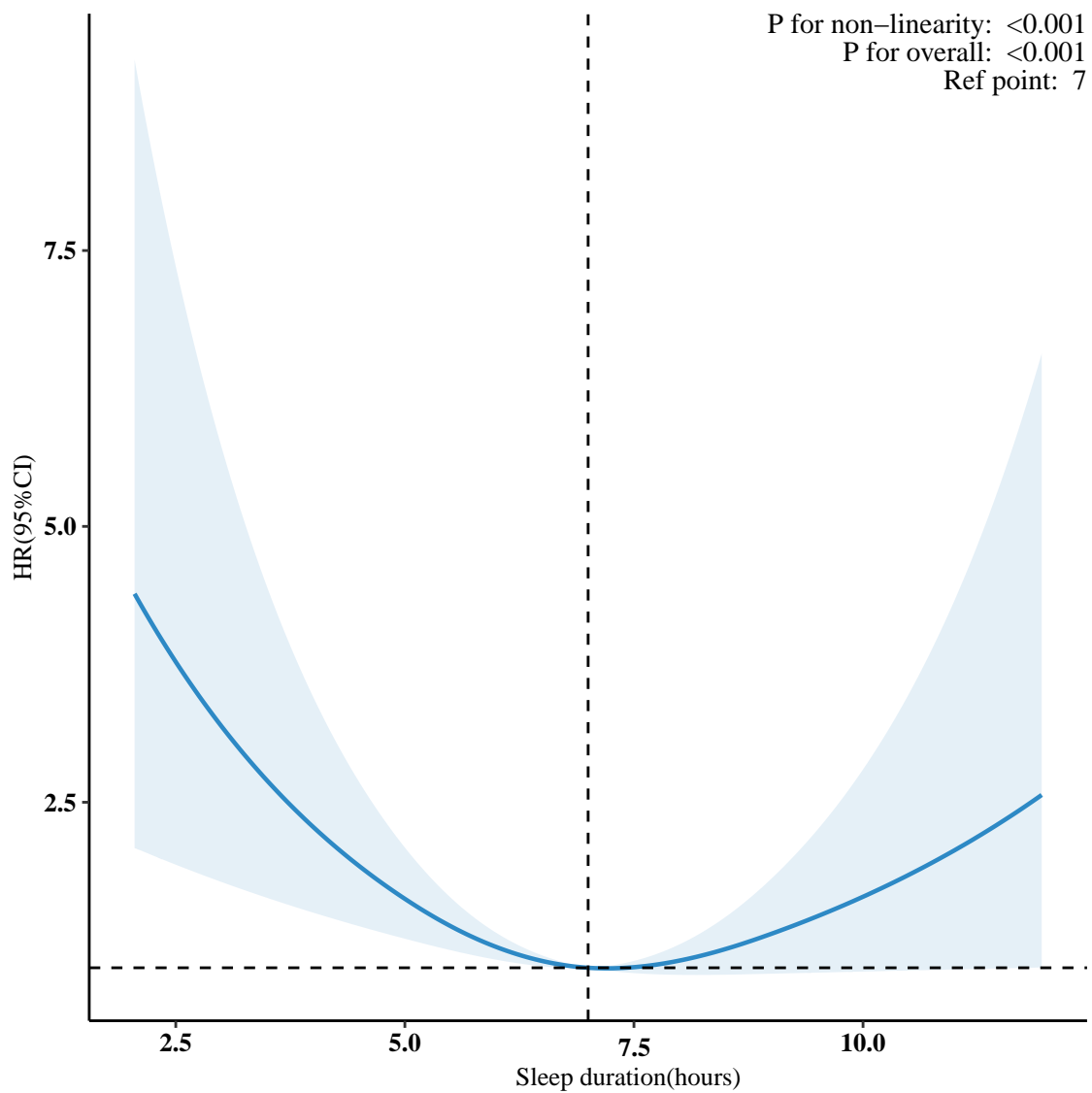

Supplement: Supplementary file 1 — Supplementary Material 1: Supplementary Figure 1: Sleep Duration Inflection Point Diagram. Abbreviations: CI, confidence interval; CVD, cardiovascular disease; HR, hazard ratio. [file 12872_2025_5252_MOESM1_ESM.pdf]

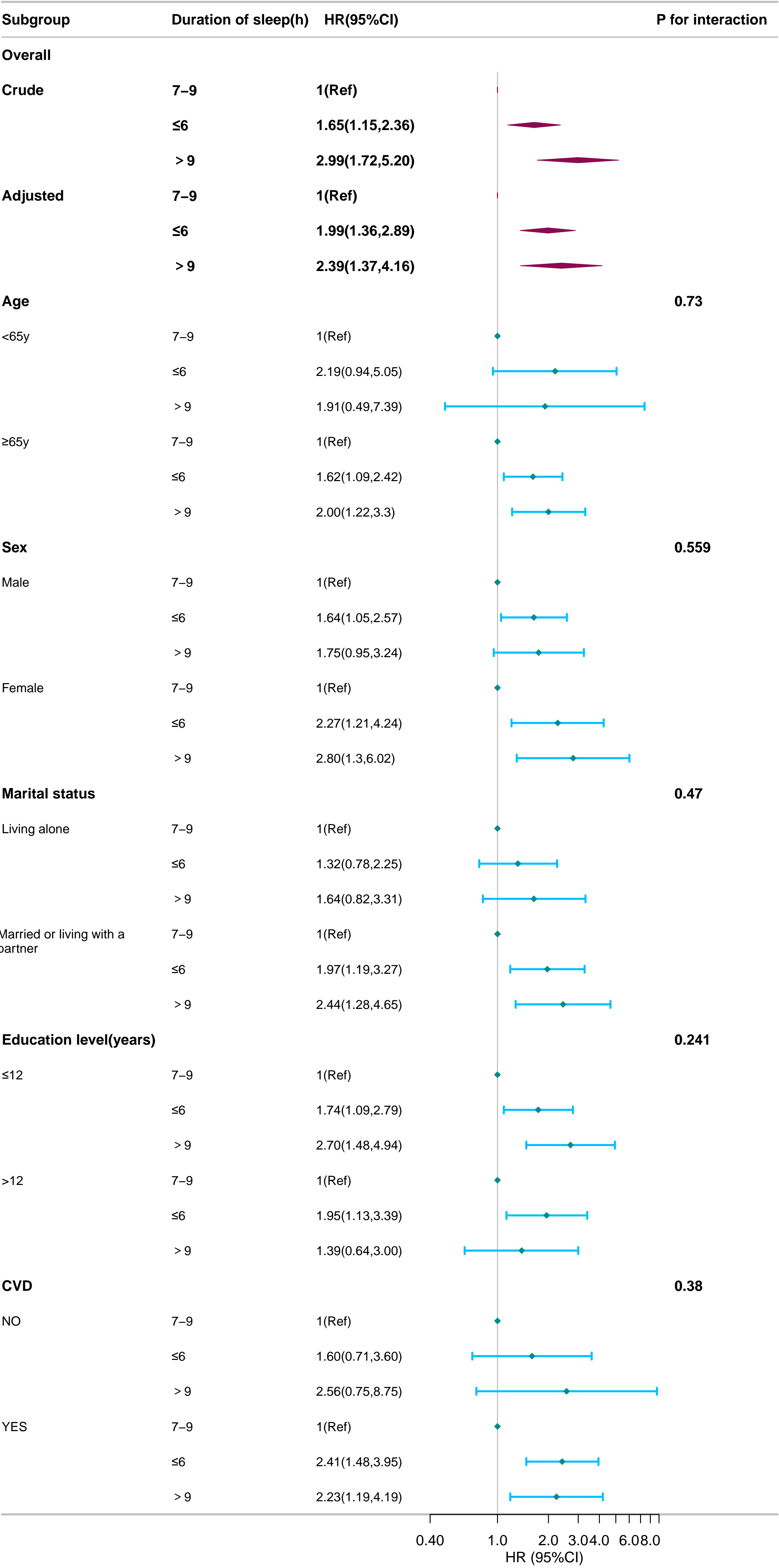

Supplement: Supplementary file 2 — Supplementary Material 2: Supplementary Figure 2：Forest plot of multivariable logistics analysis between sleep duration and CVD mortality. Note: The stratifications were adjusted for all variables（age, sex, BMI, Race/ethnicity, Education level, Marital status, Drink status, Smoking status, Vigorous recreational activities, Current health status, Chronic diseases）except for the stratification factor itself. Squares represent the HRs and horizontal lines represent 95% CIs. Diamonds represent the overall HR, and the outer points of the diamonds represent the 95% CI. Abbreviations: BMI, body mass index; CI, confidence interval; CVD, cardiovascular disease; HR, hazard ratio; NHANES, National Health and Nutrition Examination Survey. [file 12872_2025_5252_MOESM2_ESM.pdf]

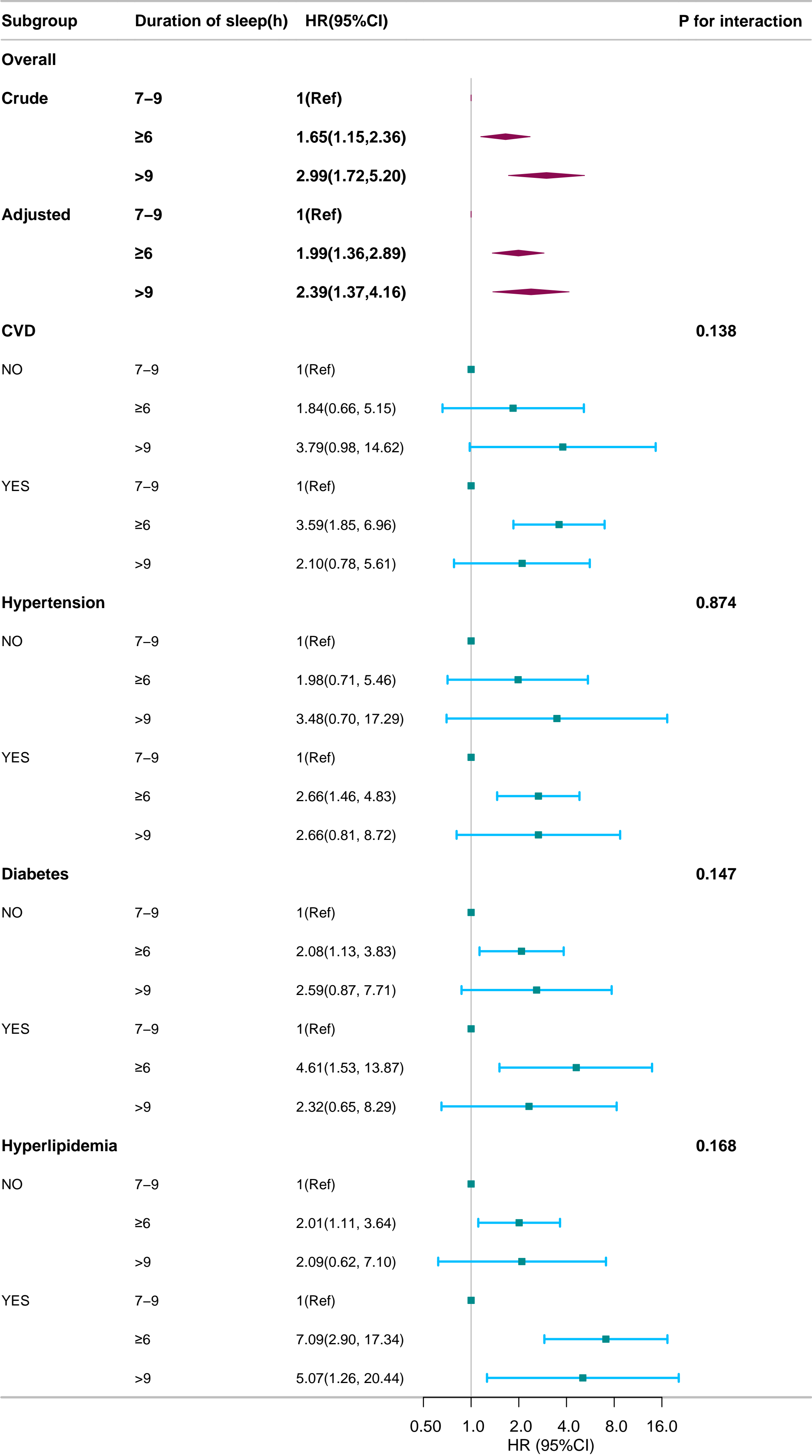

Supplement: Supplementary file 3 — Supplementary Material 3: Supplementary Figure 3:Forest plot of multivariable logistics analysis between Hazard ratios of CVD mortality by Sleep duration among. Abbreviations: HR, hazard ratio; CI, confidence interval; CVD, cardiovascular disease. [file 12872_2025_5252_MOESM3_ESM.pdf]
