# Supplementary material for: Association between sleep duration and CVD mortality: a prospective cohort study based on middle-aged and elderly chest pain patients
Source: BMC Cardiovasc Disord. 2025 Nov 26;25:846. doi: 10.1186/s12872-025-05252-z (PMC12659272; doi:10.1186/s12872-025-05252-z)
Supplement: Supplementary file 4 — Supplementary Material 4.Supplementary Table 1. Association of covariates and CVD mortality. Abbreviations: CI, confidence interval; OR, odds ratio. [file 12872_2025_5252_MOESM4_ESM.docx]

Table S1 Association of covariates and CVD mortality.

| Variable | HR (95%CI) | *P*-value |
| --- | --- | --- |
| Sex |  |  |
| Male | 1(Reference) |  |
| Female | 0.62 (0.41,0.93) | 0.022 |
| Age (years) | 1.13 (1.10,1.15) | ＜0.001 |
| BMI (kg/m^2^) | 0.96 (0.93,1.00) | 0.033 |
| Race/ethnicity |  |  |
| Mexican American | 1(Reference) |  |
| Hispanic | 0.85 (0.54,1.35) | 0.495 |
| Non-Hispanic White | 0.84 (0.47,1.49) | 0.542 |
| Non-Hispanic Black | 0.39 (0.15,0.98) | 0.046 |
| Others | 0.61 (0.25,1.47) | 0.269 |
| Education level (years) |  |  |
| ≤12 | 1(Reference) |  |
| ＞12 | 0.66 (0.45,0.98) | 0.039 |
| Marital status |  |  |
| Living alone | 1(Reference) |  |
| Married or living with a partner | 2.61 (1.82,3.75) | ＜0.001 |
| Drink status |  |  |
| No | 1(Reference) |  |
| Yes | 0.82 (0.54,1.24) | 0.349 |
| Smoking status |  |  |
| Never | 1(Reference) |  |
| Former | 0.81 (0.55,1.19) | 0.274 |
| Current | 0.92 (0.57,1.48) | 0.728 |
| Vigorous recreational activities |  |  |
| No | 1(Reference) |  |
| Yes | 0.39 (0.18,0.84) | 0.016 |
| Current health status |  |  |
| Very good to excellent | 1(Reference) |  |
| Good | 1.61 (0.94, 2.75) | 0.083 |
| Poor to fair | 2.72 (1.58,4.67) | <0.001 |
| Chronic diseases |  |  |
| No | 1(Reference) |  |
| Yes | 3.94(1.97,7.86) | <0.001 |
| CVD |  |  |
| No | 1(Reference) |  |
| Yes | 6.16(4.03,9.42) | <0.001 |
| Dyslipidemia |  |  |
| No | 1(Reference) |  |
| Yes | 0.74(0.49,1.13) | 0.164 |
| High blood pressure |  |  |
| No | 1(Reference) |  |
| Yes | 2.57(1.61,4.10) | <0.001 |
| Diabetes |  |  |
| No | 1(Reference) |  |
| Yes | 2.03(1.46,2.82) | <0.001 |
| Tumors |  |  |
| No | 1(Reference) |  |
| Yes | 1.67（1.13,2.48） | 0.011 |

Abbreviations: CI, confidence interval; OR, odds ratio.
